# The Sufficient Immunoregulatory Effect of Autologous Bone Marrow-Derived Mesenchymal Stem Cell Transplantation on Regulatory T Cells in Patients with Refractory Rheumatoid Arthritis

**DOI:** 10.1155/2020/3562753

**Published:** 2020-04-28

**Authors:** Mohsen Ghoryani, Zhaleh Shariati-Sarabi, Jalil Tavakkol-Afshari, Mojgan Mohammadi

**Affiliations:** ^1^Department of Laboratory Sciences, School of Paramedical Sciences, Torbat Heydariyeh University of Medical Sciences, Torbat Heydariyeh, Iran; ^2^Research Center of Advanced Technologies in Medicine, Torbat Heydariyeh University of Medical Sciences, Torbat Heydariyeh, Iran; ^3^Allergy Research Center, Mashhad University of Medical Sciences, Mashhad, Iran; ^4^Rheumatic Diseases Research Center, Mashhad University of Medical Sciences, Mashhad, Iran; ^5^Internal Medicine Department, Imam Reza Hospital, Mashhad University of Medical Sciences, Mashhad, Iran; ^6^Immunology Research Center, Mashhad University of Medical Sciences, Mashhad, Iran; ^7^Department of Immunology, Faculty of Medicine, Mashhad University of Medical Sciences, Mashhad, Iran

## Abstract

Rheumatoid arthritis (RA) is an advanced autoimmune disease described by joint involvement. The special properties of mesenchymal stem cells (MSCs) introduced them as a potential therapeutic candidate for RA. In this study, a single dose of autologous MSCs isolated from bone marrow (autologous BM-MSCs, 1 × 10^6^ per kg) was injected intravenously into 13 patients suffering from refractory RA who were followed up within 12 months after the intervention to evaluate immunological elements. Our results showed that the gene expression of forkhead box P3 (FOXP3) in peripheral blood mononuclear cells (PBMCs) considerably increased at month 12. We found a substantial increasing trend in the culture supernatant levels of IL-10 and transforming growth factor-beta 1 (TGF-*β*1) in PBMCs from the beginning of the intervention up to the end. Our data may reflect the sufficient immunoregulatory effect of autologous BM-MSCs on regulatory T cells in patients suffering from refractory RA.

## 1. Introduction

Rheumatoid arthritis (RA) is an advanced and systemic inflammatory autoimmune disease described by joint involvement, leading to practical debility [1]. Several environmental and genetic factors are involved in the pathogenesis of RA [2, 3]. The precise cause of RA is yet to be known, but some evidence recommends the role of T helper cell subtypes, regulatory T cells (Tregs), and their cytokines in the pathogenesis of RA [4, 5]. The disproportion between Treg/Th17 and Th1/Th2 cells appears to influence the development of RA [4, 6]. Th1 cells generate proinflammatory mediators such as tumor necrosis factor-alpha (TNF-*α*) and interferon-gamma (IFN-*γ*) [7]. Th2 cells, by producing the signature cytokine IL-4, prevent the differentiation towards Th1 and Th17 cells [5], the latter being a key mediator in inflammatory cellular immune responses that generate high levels of IL-17 [8]. Tregs suppress pathological autoreactive immune responses by producing IL-10 and transforming growth factor-beta (TGF-*β*) [9].

Patients with RA are commonly treated with disease-modifying antirheumatic drugs (DMARDs) and biological agents that may entail side effects and gradually render the subjects resistant to the medications [10, 11]. The special properties of mesenchymal stem cells (MSCs) such as multipotent and immunomodulatory capacities have introduced them as potential therapeutic candidates for RA. MSCs modulate B and T cell proliferation and suppress inflammatory processes associated with RA [12–14].

In this research, we evaluated the changes in immunological factors of patients suffering from refractory RA following treatment with autologous MSCs isolated from the bone marrow (autologous BM-MSCs).

## 2. Materials and Methods

### 2.1. Patients

In this single-arm clinical trial, 13 patients with refractory RA receiving the maximum approved dose of conventional DMARDs were recruited from July 2016 to October 2017 at Imam Reza Hospital, Mashhad, Iran, according to the inclusion criteria previously described [15]. All patients provided informed consent to register in the clinical trial. For details about the ethical approval and clinical trial registration, please refer to our previous study [15].

### 2.2. MSC Transplantation

Autologous BM-MSC preparation and transplantation for the 13 patients with refractory RA were performed by our team according to the protocol delineated in our recent publication [15]. A single dose of autologous BM-MSCs (1 × 10^6^ per kg of body weight) was injected intravenously in all 13 patients who were followed up at 1, 6, and 12 months after the intervention to evaluate immunological factors.

### 2.3. Laboratory Methods

#### 2.3.1. Cell Culture

Peripheral blood mononuclear cells (PBMCs) were isolated by Lymphocyte®-H (Cedarlane, Canada) and adjusted to 2 × 10^6^ cells/ml in a RPMI1640 medium (Caisson, USA) consisting of 1% Pen/Strep (Caisson) and 10% fetal bovine serum (Gibco, USA). PBMCs were then cultured in polystyrene plates (SPL, South Korea) with phytohaemagglutinin (PHA, 5 *μ*g/ml) (Biowest, USA), which were put for incubation for 18 hours, in a humidified CO_2_ incubator at 37°C to assess the gene expression levels of transcription factors, and 72 hours to evaluate cytokine production.

#### 2.3.2. RNA Extraction and cDNA Preparation

Total RNA extraction was done using a Total RNA Purification Mini kit (Favorgen, Taiwan), and cDNA was then synthesized by a cDNA Synthesis Kit (Favorgen), according to the manufacturer's information.

#### 2.3.3. SYBR® Green Real-Time PCR

Forward and reverse primers for forkhead box P3 (FOXP3), GATA binding protein 3 (GATA3), T-bet and retinoic acid-related orphan receptor gamma t (ROR-*γ*t) as the target genes, and glyceraldehyde-3-phosphate dehydrogenase (GAPDH) as the housekeeping gene were developed in-house employing Beacon Designer 7.9 (Premier Biosoft International, USA) (Table 1); next, they were blasted at the NCBI website (Primer-BLAST) to check the specificity. Real-time PCR was carried out on cDNA samples in a Rotor-Gene 6000 real-time analyzer (QIAGEN, Germany) using SYBR® Premix EX Taq II (2x) (Takara Bio Inc., Japan). Details of PCR conditions would be available upon request. Expression of transcription factors at mRNA levels were calculated employing the 2^-*ΔΔ*CT^ method and presented as a relative expression related to before mesenchymal stem cell transplantation (MSCT).

#### 2.3.4. Cytokine Analysis

The levels of TNF-*α*, IFN-*γ*, IL-10, IL-4, and IL-17A in a supernatant of PBMCs cultured in the presence of PHA were determined by commercially obtainable enzyme-linked immunosorbent assay (ELISA) kits (BioLegend, USA). The ELISA kit for TGF-*β* was purchased from Cloud-Clone Corp., USA, to detect this cytokine in culture supernatants. The ELISA procedures for all cytokines were carried out following the manufacturer's information. The detection limit was >2 pg/ml for TNF-*α*, IL-4, IL-10, and IL-17A; >4 pg/ml for IFN-*γ*; and >5.8 pg/ml for TGF-*β*.

### 2.4. Statistical Analysis

For comparing pre- and postintervention findings regarding the immunological and clinical factors, the generalized estimating equation (GEE) analytical method was employed. Pearson's correlation analysis was performed to analyze the correlation between the disease activity score 28-erythrocyte sedimentation rate (DAS28-ESR) and supernatant levels of cytokines. All analyses were done with IBM SPSS Statistics 21 (IBM Corp, USA). *P* value < 0.05 was defined as statistically significant.

## 3. Results

### 3.1. Demographic Data for Patients with Refractory RA

Thirteen female patients suffering from refractory RA at a mean ± SD age of 44 ± 7.50 years were registered in this trial. The patients had a mean ± SD disease duration of 12.16 ± 4.08 years. DAS28-ESR decreased significantly at 1, 6, and 12 months as compared to before MSCT (mean ± SEM, 5.56 ± 0.40 before MSCT to 5.04 ± 0.44 at 1 month, *P* < 0.001; to 5.06 ± 0.34 at 6 months, *P* < 0.05; and to 4.72 ± 0.50 at 12 months, *P* < 0.001).

### 3.2. Gene Expression Analysis

**Figure 1:**
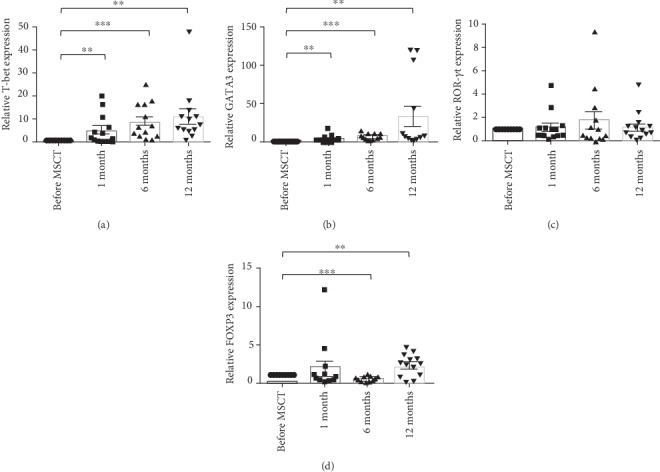
Expression of transcription factors at mRNA levels in 13 patients with refractory RA before and after MSCT. Data are presented as mean ± SEM relative expression related to before MSCT. Relative mRNA levels before MSCT were set to 1: (a) T-bet; (b) GATA3; (c) ROR-*γ*t; (d) FOXP3. ^∗∗^*P* < 0.01 and ^∗∗∗^*P* < 0.001. RA: rheumatoid arthritis; MSCT: mesenchymal stem cell transplantation; GATA3: GATA binding protein 3; ROR-*γ*t: retinoic acid-related orphan receptor gamma t; FOXP3: forkhead box P3; SEM: standard error of the mean.

The T-bet mRNA expression showed a significant increasing trend after MSCT (mean ± SEM, 1.00 ± 0.0 before MSCT, 5.64 ± 1.80 at 1 month, 9.31 ± 2.12 at 6 months, and 11.48 ± 3.26 at 12 months) (Figure 1(a)). Similar to the T-bet mRNA expression pattern, GATA3 mRNA expression showed a significant increasing trend after MSCT (mean ± SEM, 1.00 ± 0.0 before MSCT, 4.60 ± 1.41 at 1 month, 8.24 ± 1.18 at 6 months, and 34.58 ± 13.36 at 12 months) (Figure 1(b)). No statistically significant change was observed in the ROR-*γ*t mRNA expression following MSCT (mean ± SEM, 1.00 ± 0.0 before MSCT, 1.24 ± 0.35 at 1 month, 1.78 ± 0.72 at 6 months, and 1.25 ± 0.34 at 12 months) (Figure 1(c)). FOXP3 mRNA expression was significantly reduced at 6 months after MSCT. A significant increase was found in FOXP3 mRNA expression at 12 months after MSCT (mean ± SEM, 1.00 ± 0.0 before MSCT, 1.87 ± 0.91 at 1 month, 0.51 ± 0.10 at 6 months, and 2.27 ± 0.40 at 12 months) (Figure 1(d)).

### 3.3. Cytokine Analysis

**Figure 2:**
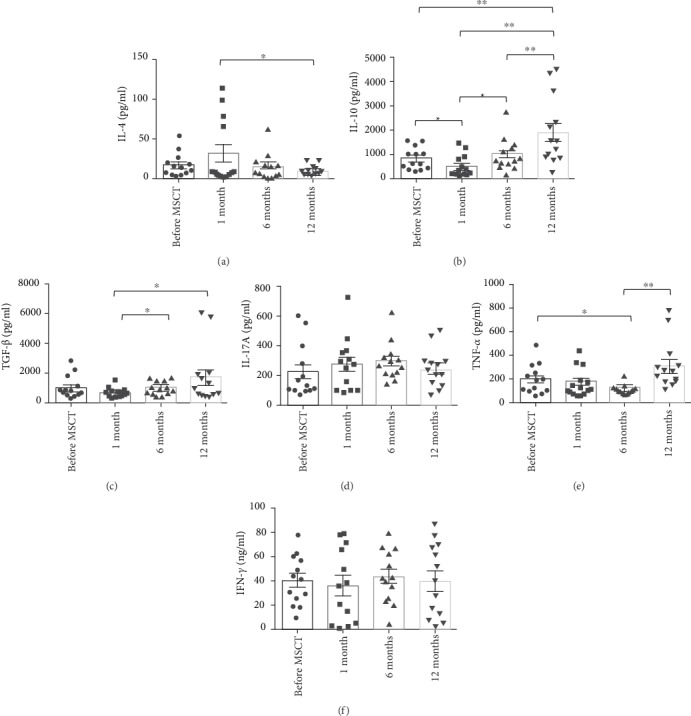
Supernatant levels of cytokines before and after MSCT: (a) IL-4; (b) IL-10; (c) TGF-*β*; (d) IL-17A; (e) TNF-*α*; (f) IFN-*γ*. Data are presented as mean ± SEM. ^∗^*P* < 0.05 and ^∗∗^*P* < 0.01. MSCT: mesenchymal stem cell transplantation; TGF-*β*: transforming growth factor-beta; TNF-*α*: tumor necrosis factor-alpha; IFN-*γ*: interferon-gamma; SEM: standard error of the mean.

Supernatant levels of IL-4 were not significantly altered following MSCT as compared to pre-MSCT. A substantial decrease in supernatant levels of IL-4 was detected at 12 months when compared to 1 month (mean ± SEM, 17.07 ± 4.17 pg/ml before MSCT, 31.44 ± 11.50 pg/ml at 1 month, 15.37 ± 4.58 pg/ml at 6 months, and 8.69 ± 1.74 pg/ml at 12 months) (Figure 2(a)).

Supernatant levels of IL-10 were significantly reduced 1 month following MSCT and displayed a substantial increase at 12 months as compared to pre-MSCT. Further seen was a considerable augment in the supernatant levels of IL-10 at 6 months as compared to 1 month. Additionally, supernatant levels of IL-10 significantly increased at 12 months when compared to 1 month and 6 months (mean ± SEM, 824.88 ± 125.42 pg/ml before MSCT, 522.61 ± 123.92 pg/ml at 1 month, 1014.84 ± 184.07 pg/ml at 6 months, and 1897.97 ± 388.19 pg/ml at 12 months) (Figure 2(b)).

No significant variation was found in the supernatant levels of TGF-*β* following MSCT vs. prior to MSCT. A substantial increase was seen in supernatant levels of TGF-*β* at 12 months as compared to 1 month. Moreover, the supernatant levels of TGF-*β* displayed a substantial increase at 6-month vs. 1-month follow-up (mean ± SEM, 1063.42 ± 206.25 pg/ml before MSCT, 670.95 ± 98.22 pg/ml at 1 month, 1035.16 ± 133.65 pg/ml at 6 months, and 1699.76 ± 531.78 pg/ml at 12 months) (Figure 2(c)).

No statistically significant discrepancy was detected in the supernatant levels of IL-17A (mean ± SEM, 226.63 ± 50.00 pg/ml before MSCT, 277.20 ± 54.57 pg/ml at 1 month, 299.20 ± 38.66 pg/ml at 6 months, and 245.24 ± 35.13 pg/ml at 12 months) and IFN-*γ* (mean ± SEM, 40.46 ± 5.61 ng/ml before MSCT, 36.02 ± 8.37 ng/ml at 1 month, 43.74 ± 6.10 ng/ml at 6 months, and 40.00 ± 8.38 ng/ml at 12 months) following MSCT (Figures 2(d) and 2(f)).

Supernatant levels of TNF-*α* were significantly reduced 6 months after MSCT. No substantial variation was found in the supernatant levels of TNF-*α* at 1 month and 12 months vs. before MSCT. Additionally, the supernatant levels of TNF-*α* showed a significant increase at 12 months vs. 6 months (mean ± SEM, 203.40 ± 37.44 pg/ml before MSCT, 181.64 ± 32.29 pg/ml at 1 month, 123.72 ± 14.39 pg/ml at 6 months, and 311.25 ± 61.52 pg/ml at 12 months) (Figure 2(e)).

### 3.4. Correlation Analysis

**Figure 3:**
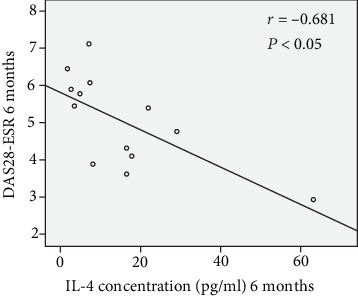
Negative significant correlation between DAS28-ESR and supernatant levels of IL-4 at 6-month follow-up. DAS28-ESR: disease activity score 28-erythrocyte sedimentation rate.

Correlation analysis revealed a significant negative relationship between DAS28-ESR and supernatant levels of IL-4 at 6-month follow-up (Figure 3). No significant correlation was found between any of the other cytokines and DAS28-ESR at any of the follow-up time points.

### 3.5. Adverse Events

No negative events were observed during the 12 months of follow-up in any of the 13 patients.

## 4. Discussion

### 4.1. Gene Expression Levels

In the current research, we found a similar significant increasing tendency for both T-bet and GATA3 mRNA expression levels after MSCT. ROR-*γ*t mRNA expression levels exhibited no substantial discrepancy following treatment with MSCs. FOXP3 mRNA expression levels were reduced at 6-month follow-up, showing a substantial rise at 12 months after MSCT.

To our knowledge, there is limited information available regarding the effects of MSCT on the expression of immune-related genes in autoimmune diseases. There is only one publication concerning the influence of MSCT on the gene expression pattern of immune-related transcription factors in the autoimmune diseases, reported by Mohyeddin Bonab et.al. [16] who observed no remarkable change in FOXP3 mRNA expression levels following the intrathecal administration of autologous BM-MSCs in patients suffering from multiple sclerosis (MS).

Previous studies have reported that T-bet enhances the differentiation towards Th1 cells whereas inhibiting the differentiation of Th2 cells [17]. GATA3 acts contrary to T-bet and stimulates the differentiation of Th2 cells by preventing Th1 cell differentiation [18]. FOXP3 regulates differentiation and immunoregulatory features of Tregs, whose survival depends on IL-2 [19]. ROR-*γ*t is involved significantly in the differentiation towards Th17 cells [18]. FOXP3 plays an important role in the expression of the alpha subunit of IL-2 receptor (CD25) on the cell surface of Tregs. GATA3 enhances the expression of CD25 by increasing the expression of FOXP3 in Tregs and conduces to maintaining the activities of Tregs [19–21]. In addition to T-bet, GATA3, ROR-*γ*t, and FOXP3, T cell differentiation into distinct subtypes requires the collaboration of other transcription factor families such as run-related transcription factor (RUNX) and signal transducer and activator of transcription (STAT) [18]. For instance, the RUNX3-T-bet complex enhances the expression of IFN-*γ*, promoting Th1 cell differentiation. GATA3 binding to RUNX3 inhibits the enhancing effects of RUNX3 on the generation of IFN-*γ* which consequently ends in the impairment of Th1 differentiation [22]. On the other hand, it should be borne in mind that posttranscriptional factors such as microRNAs and RNA-binding proteins (RBPs) strongly affect protein levels in cells [23]. MicroRNA-155 and microRNA-146a are two known microRNAs whose regulatory effects on the differentiation of T cell subtypes have been investigated [24].

The influence of pharmacotherapy on the gene expression levels have been further researched in the previous studies [25, 26]. Therefore, mRNA expression levels of ROR-*γ*t and other transcription factors in our study might be influenced by medications taken by patients with refractory RA who underwent MSCT.

Regardless of posttranscriptional mechanisms and effects of conventional medications on mRNA expression levels, our data show that autologous BM-MSCs in RA may provide a suitable condition for the differentiation towards both Th1 and Th2 cells. Further studies on the influence of MSCT on microRNAs and transcription factors linked to the differentiation of T cell subtypes not only in the gene expression levels but also in the protein levels may be conducive to a better evaluation of the immunoregulatory mechanisms of MSCs in autoimmune diseases such as RA.

### 4.2. Levels of Cytokines in Supernatants

In our study, supernatant levels of IL-4 showed a decreasing trend from 1 month to the end of the study. Contrary to IL-4, we detected a significant increasing trend in the supernatant levels of TGF-*β* and IL-10 from the 1-month to 12-month follow-ups. Supernatant levels of TNF-*α* were significantly reduced at 6 months after MSCT. We found no substantial discrepancy concerning the supernatant levels of IFN-*γ* and IL-17A following MSCT.

Wang et.al stated considerable decrease in the levels of TNF-*α* in the supernatants of activated PBMCs at 1 week and 3 months following intravenous injection of allogeneic MSCs isolated from the umbilical cord (allogeneic UC-MSCs) into patients with refractory RA [27]. Further in agreement with our findings, Wang et.al reported a substantial augment in the serum levels of TGF-*β* and a noticeable reduction in the serum levels of TNF-*α*. Moreover, they reported no remarkable variation in the serum levels of IL-17A following the intravenous injection of allogeneic UC-MSCs into patients suffering from active and refractory systemic lupus erythematosus (SLE) [28]. In contrast to our findings, Mohyeddin Bonab et.al observed no substantial change in the serum levels of IL-4, IL-10, and TGF-*β* during the 12-month follow-up after intrathecal administration of autologous BM-MSCs in patients suffering from MS [16].

IL-4, as an essential cytokine for the differentiation of Th2 cells, is mostly produced by these T cell subtypes [29]. Monocytes, macrophages, Th2 cells, and Tregs are the main producers of IL-10 which is an anti-inflammatory cytokine inhibiting the synthesis of IL-1*β*, IL-8, and TNF-*α*. IL-10 downregulates the surface expression of HLA-DR and B7 costimulatory molecules on antigen presenting cells of the synovial fluid which further impairs T cell activation [30]. Tregs and macrophages are the major sources of TGF-*β* which inhibits the differentiation towards Th1 cells by inhibiting T-bet and reducing IL-12 receptor expression. In the presence of IL-6, TGF-*β* stimulates the expression of ROR-*γ*t and suppresses FOXP3 expression, resulting in the differentiation towards Th17 cells [30, 31]. IL-17 is predominantly secreted by Th17, CD8+ T cells, and neutrophils. IL-17 stimulates the production of inflammatory mediators such as IL-6 and TNF-*α*. Elevated levels of IL-17 have been reported in the serum and synovial fluid of patients with RA [7, 32]. TNF-*α* is a critical cytokine responsible for inflammation in RA. Monocytes, macrophages, natural killer (NK) cells, and mastocytes are the main producers of TNF-*α*. Patients with RA show increased levels of TNF-*α* in the synovial fluid [32]. IFN-*γ* is an effective cytokine regarding the differentiation of Th1 cells which along with CD8+ T cells and NK cells are known as the main producers of IFN-*γ*, an important activator of macrophages [18, 33].

Given the crucial role of Th2 cells in humoral immunity and production of antibodies [18], the decreasing trend in the supernatant levels of IL-4, as essential cytokines for the differentiation of Th2 cells, following MSCT indicates that MSCs may impair the production of autoantibodies in refractory RA. The negative correlation between DAS28-ESR and supernatant levels of IL-4 at month 6 may indicate the anti-inflammatory effects of IL-4 resulting in reduced disease activity score in the middle of our trial. Our findings suggest that the supernatant levels of IL-17A and IFN-*γ* might not be affected by MSCT. Additionally, the production of these cytokines by PBMCs may also be affected by conventional therapy in our patients with refractory RA. The increasing trend in the supernatant levels of TGF-*β* and IL-10 and the decreasing pattern in the supernatant levels of TNF-*α* following MSCT might be elucidated by the immunoregulatory effects of MSCT.

## 5. Conclusions

The present data indicated that MSC therapy in patients with refractory RA has significant immunomodulatory effects, especially through increasing the protein levels of IL-10 and TGF-*β*, as two important cytokines of Tregs. Moreover, the substantial increase in the gene expression of FOXP3 as a unique transcription factor of Tregs at the end of the intervention may reflect the ability of MSCs to differentiate T lymphocytes towards Tregs in the immune system. A control group including patients suffering from refractory RA who just received conventional therapies without MSCT might help us for a better understanding regarding the potential of such therapies and their interference effects on MSCT for future works. Moreover, the increase in the dose of MSCs and/or replication of injections might maintain the immunomodulatory effects of MSCs in patients suffering from refractory RA, a point to be kept in mind for future research.

## Figures and Tables

**Table 1:** Primer sequences designed for SYBR® Green real-time PCR.

Gene name	Accession number	Sequence
T-bet-F	NM_013351.1	5′-ATTGCCGTGACTGCCTACCAGA-3′
T-bet-R		5′-GGAATTGACAGTTGGGTCCAGG-3′
GATA3-F	NM_001002295.1	5′-ACCACAACCACACTCTGGAGGA-3′
GATA3-R		5′-TCGGTTTCTGGTCTGGATGCCT-3′
ROR-*γ*t-F	NM_005060.4	5′-CCCTGACAGAGATAGAGCACC-3′
ROR-*γ*t-R		5′-TTCCCACATCTCCCACATGG-3′
FOXP3-F	NM_014009.3	5′-GGCACAATGTCTCCTCCAGAGA-3′ 5′-CAGATGAAGCCTTGGTCAGTGC-3′
FOXP3-R		
GAPDH-F	NM_001289746.1	5′-CACTAGGCGCTCACTGTTCTC-3′
GAPDH-R		5′-CCAATACGACCAAATCCGTTGAC-3′

Note: F: forward primer; R: reverse primer; GATA3: GATA binding protein 3; ROR-*γ*t: retinoic acid-related orphan receptor gamma t; FOXP3: forkhead box P3; GAPDH: glyceraldehyde-3-phosphate dehydrogenase.

## Data Availability

The data used to support the findings of this study are available from the corresponding author upon request.
